# Unpacking Organic Food Purchase Intentions in an S‐O‐R Framework: The Multidimensional Moderating Roles of Consumer Knowledge and Trust

**DOI:** 10.1002/fsn3.70447

**Published:** 2025-06-19

**Authors:** Yusuf Arslan

**Affiliations:** ^1^ Business School Sakarya University Sakarya Türkiye

**Keywords:** consumer trust, environmental concerns, health consciousness, organic food, organic knowledge, S‐O‐R

## Abstract

This study applies the Stimulus‐Organism‐Response (S‐O‐R) model to examine consumers' purchase intentions for organic food. The model incorporates three stimulus (S) variables—price perceptions, health consciousness, and environmental concerns—along with the organism (O) variable of attitudes, culminating in the response (R) of purchase intention. Using structural equation modeling (SEM) on data from 474 organic consumers in Turkey, the study also investigates four moderating variables: subjective and objective knowledge and trust in organic food and labeling processes. These moderators address the commonly observed attitude‐behavioral intention gap. The results reveal that health consciousness, price perception, and environmental concerns positively influence attitudes, enhancing purchase intentions. Moreover, three out of the four moderators were found to enhance the relationship between attitudes and purchase intentions significantly. However, no evidence supported the moderating role of subjective knowledge. These findings offer valuable insights for researchers exploring consumer behavior and practitioners designing marketing strategies, emphasizing the critical role of knowledge and trust in fostering organic food adoption in emerging markets.

## Graphical abstract

This study employs the Stimulus‐Organism‐Response (S‐O‐R) model, revealing that health consciousness, environmental concerns, and price perception positively influence consumer attitudes towards organic foods, significantly enhancing purchase intentions. Trust in organic foods, trust in labeling processes, and objective knowledge significantly strengthen the relationship between attitudes and purchase intentions. Subjective knowledge, however, does not moderate this relationship, emphasizing the critical role of factual knowledge and trust in bridging the attitude‐behavioral intention gap in emerging markets.

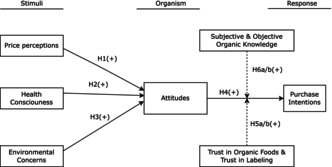

## 1 Introduction

Previous research indicates that organic foods are more nutritious and contain lower levels of pesticides and chemical residues than conventional foods (Barański et al. 2014). This trend has catalyzed a growing demand for organic food produced using healthy and sustainable methods (FiBL and IFOAM 2021). The underlying reason is an increasing number of consumers who place a premium on healthier and environmentally friendly practices in food production, with particular emphasis on organic farming (European Commission 2021). As a result, global demand for organic food has risen (Liang et al. 2017; Tariq et al. 2019).

This increasing demand became particularly pronounced during the coronavirus pandemic. During the COVID‐19 lockdown, consumption patterns shifted towards healthier food habits as consumers sought to maintain good nutritional status and reduce health risks. Consequently, this further accelerated the adoption and consumption of organic food products (Di Renzo et al. 2020). For example, organic farmlands expanded by 1.6% (1.1 million hectares) globally during 2018–2019, with the global organic market reaching €106 billion and involving more than 3.1 million organic producers (FiBL and IFOAM 2021).

Turkey presents a unique opportunity for organic market expansion due to its increasing organic production levels (Republic of Turkey Ministry of Agriculture and Foresty 2021). As the country with Europe's largest organic farming area, Turkey contributed 8.1% of the 3.3 million tons of organic imports to Europe in 2018 (Willer and Lernoud 2018). However, despite this productive capacity, domestic organic food retail sales in Turkey still lag significantly behind those of European countries. In 2021, per capita expenditure on organic food product consumption was approximately €1, making Turkey one of the lowest‐spending nations in Europe. Denmark's per capita organic food expenditure was €344, and Austria's was €216 in the same period (FiBL and IFOAM 2021). This stark contrast highlights Turkey's untapped potential in organic food consumption.

Nevertheless, empirical studies on Turkish consumers' organic food decision‐making and purchasing behavior remain limited, creating an obstacle to unlocking this potential. As with emerging economies in general, a lack of understanding of consumer behavior hinders the realization of Turkey's organic market potential (Basha and Lal 2019). Additionally, sociocultural influences on food choices further underscore the need for market‐specific insights (Monterrosa et al. 2020). Against this backdrop, the overall purpose of this study is to provide further empirical insights into the key antecedents of Turkish consumers' organic food purchasing behavior. Accordingly, one of the primary purposes of this study is to empirically examine how Turkish consumers' attitudes towards organic food are shaped by both symbolic motives (e.g., health and environmental benefits) and economic motives (e.g., price) (Honkanen et al. 2006; Arvola et al. 2008). Additionally, we examine how those attitudes affect consumers' purchase intentions for organic foods. Thus, we pose the following two research questions:

*RQ1: How do price perceptions, health consciousness, and environmental concerns affect consumer attitudes towards organic foods?*

*RQ2: How do attitudes affect consumer purchase intentions towards organic foods?*

One notable concern in this context pertains to consumers' lack of accurate knowledge about organic products. Many empirical studies in the field measure organic product knowledge only subjectively (Pacho 2020; Eberle et al. 2022). Therefore, the impact of actual knowledge on purchasing behavior has not been fully understood. A similar issue applies to consumer trust, a crucial factor influencing organic food choices (Hamzaoui‐Essoussi et al. 2013). Prior studies have often examined trust from a single perspective, such as trust in labeling (Liang and Lim 2020) or trust in retailers (Yu et al. 2021), even though trust is a multidimensional concept that encompasses confidence in both producers/retailers and organic certification processes (Nuttavuthisit and Thøgersen 2017). This lack of attention to the multifaceted nature of trust, along with the limited consideration of objective knowledge, represents a critical gap in the literature. Additionally, a meta‐analysis by Massey et al. (2018) highlighted a gap in the sustainable food literature regarding the relationship between attitudes and behavioral intentions. By introducing understudied moderators, specifically the two dimensions of trust and both subjective and objective knowledge, we aim to address these gaps. In this framework, the third research question is formulated as follows:

*RQ3: How do subjective and objective knowledge, as well as trust in organic foods and trust in the labeling process, moderate the relationship between attitudes and purchase intention?*

By addressing these questions, this study aims to advance sustainable consumption research and provide actionable insights for stakeholders in Turkey's organic food market, ultimately contributing to the organic food literature in emerging markets.

## 2 Theoretical Background and Hypotheses Development

### 2.1 S‐O‐R Model

The conceptual framework of this study builds upon the S‐O‐R model (Mehrabian and Russell 1974), which is rooted in environmental psychology and widely used in consumer behavior research, including studies on organic food consumption (e.g., Liang and Lim 2020; Tandon et al. 2021; Talwar et al. 2021; Xiang and Liu 2024). It is argued that the S‐O‐R theory provides a suitable theoretical infrastructure for explaining consumers' purchase intentions and stated buying behaviors towards organic products (Talwar et al. 2021). Furthermore, given the critical influence of environmental factors on consumer behavior, the SOR model provides a structured framework for assessing how environmental stimuli influence consumers' psychological parameters (e.g., perception and attitude) (Cao et al. 2023). The model posits that environmental stimuli (S) affect individuals' internal cognitive and affective reactions (O), leading to response behaviors (R). According to Jacoby (2002), “stimulus consists of the environment as encountered by the individual at a particular moment in time,” which can include factors like pricing, product packaging, or store atmosphere. Health‐related factors can also function as stimuli, given rising anxiety and safety concerns related to food consumption that have been linked to organic food purchase motivations (Kareklas et al. 2014; Pham et al. 2019). According to Liang and Lim (2020), organism factors are the psychological processes, both conscious and unconscious, that a person undergoes in response to external stimuli. These include values, attitudes, cognitive networks, personality traits, and emotions. The outcome is the response, which can either be an avoidance behavior or an approach behavior (Lee and Yun 2015).

Recent organic food studies have operationalized stimuli through various variables such as organic food values (Sultan et al. 2021), corporate image (Yu et al. 2021), health consciousness (Talwar et al. 2021; Morais et al. 2024), food safety concern (Talwar et al. 2021), and self‐care (Laos‐Espinoza et al. 2024). Organism factors in prior work include attitudes (Sultan et al. 2021; Laos‐Espinoza et al. 2024), trust (Yu et al. 2021), and personal traits like openness to change and self‐identity (Talwar et al. 2021). Response variables primarily focus on behavioral intentions (Yu et al. 2021; Sultan et al. 2021; Talwar et al. 2021; Laos‐Espinoza et al. 2024) or actual purchasing behavior (Tandon et al. 2021).

In this study, we assess the effects of consumers' health consciousness, environmental concerns, and price perceptions of organic food (S) as antecedents of attitudes (O), which in turn affect the response to purchase intention (R). There are specific reasons to focus on these elements in this study. Firstly, although it is a widely studied critical factor, the literature is mixed on the effect of environmental concern on attitudes towards organic products. For example, Yadav and Pathak (2016) reported a positive effect on Indian consumers, while Eberle et al. (2022) found similar results among Brazilian consumers, which aligns with the existing literature. In contrast, Yilmaz and Ilter (2017) found no significant effect among Turkish consumers, as did Pham et al. (2019) and Le‐Anh and Nguyen‐To (2020) among Vietnamese consumers, which they attributed to limited awareness of the environmental benefits of organic products. The authors suggest that environmental impact may not be the primary motive for purchasing organic products in developing countries. The main reason for selecting environmental concerns as a stimulus in this study is the inconsistent results regarding the impact of this widely studied and important concept on attitudes towards organic products, particularly within the context of developing countries.

A recent meta‐study revealed a gap between attitudes towards organic products and purchase intentions (Massey et al. 2018). However, the relationship between these concepts has yielded mixed results. For example, while Nedra et al. (2015) and Hempel and Hamm (2016) report no significant effect, the effect is positive in studies conducted by Yilmaz and Ilter (2017), Pham et al. (2019), Nautiyal and Lal (2022), and Nguyen et al. (2023). Finally, Pellegrini and Farinello (2009) and Lee and Yun (2015) find a negative effect. These results provide us with a rationale for re‐establishing this relationship.

Finally, findings on the influence of health consciousness on attitudes have likewise been inconsistent. While Rana and Paul (2020) and Pham et al. (2019) revealed a positive effect, Nedra et al. (2015) and Yilmaz and Ilter (2017) reported an insignificant relationship. In their meta‐analysis, Rana and Paul (2020) concluded that health‐related factors as determinants of organic food purchase and attitudes need to be further explored as they yield inconsistent results in both developed and developing country contexts. This study aims to address these inconsistencies by integrating the above factors into the S‐O‐R framework and examining them systematically. By doing so, we seek to provide a more nuanced understanding of organic food purchase intentions, thereby contributing to the sustainable consumption literature.

In addition, this study builds on the S‐O‐R model and further develops its theoretical scope. In particular, considering dual conceptualizations of trust (product‐level and certification‐level) and knowledge (subjective and objective) has enabled a more in‐depth analysis of the impact of these variables on the relationship between attitude and intention. While these concepts are mostly examined in a one‐dimensional manner in existing literature, our two‐fold approach reveals the conditions under which positive attitudes are more likely to lead to purchase intentions. Thus, this study offers an original theoretical framework to re‐evaluate the frequently reported attitude‐intention gap, particularly in emerging markets where institutional trust and consumer knowledge are limited.

### 2.2 Price Perceptions of Organic Foods

In the literature, relatively high prices of organic food have been revealed as a significant obstacle to buying these products (Hughner et al. 2007; Aertsens et al. 2011). However, high prices have also been reported to create a perception of exclusivity as consumers tend to associate them with high‐quality products (Pellegrini and Farinello 2009). Hu et al. (2024) found that the influence of organic food prices on consumer purchasing behavior can vary by context. In some cases, high prices discourage purchases due to affordability concerns, while in others, they positively influence attitudes by signaling superior quality and exclusivity.

In the Turkish context, organic food prices are perceived as high, as reported in previous studies (Eryılmaz et al. 2015; Canarslan and Uz 2019). Despite the financial burden this poses for specific consumer segments, we argue that the perception of high prices could enhance positive attitudes toward organic foods by reinforcing their image as premium, high‐quality products. Thus, we propose the following hypothesis.

*Price perceptions of organic foods positively affect consumer attitudes*.

### 2.3 Health Consciousness

Prior studies indicate that health‐conscious individuals are more inclined to consume healthier food compared to those with lower levels of health consciousness (e.g., Devcich et al. 2007; Mai and Hoffmann 2012). Health consciousness can also motivate consumers' decisions to purchase organic food because many consumers believe organic food is better for their health, and this belief fosters positive attitudes. Numerous studies have found that higher health consciousness leads to more favorable attitudes towards organic foods (Rana and Paul 2020; Pham et al. 2019; Bazhan et al. 2024; Hu et al. 2024). In accordance with this broad consensus in the literature, we propose health consciousness (HC) as an important antecedent in organic food preferences. Therefore, we hypothesize:

*Health consciousness positively affects consumer attitudes towards organic foods*.

### 2.4 Environmental Concerns

In this study, we define environmental concerns as the extent to which consumers' care for environmental well‐being in their consumption activities. The literature suggests that consumers who care about the environment are more likely to buy products that do not harm the environment and that respect animal rights in production (Harper and Makatouni 2002). Product features associated with environmental friendliness motivate consumers to buy organic foods (Padel and Foster 2005). Environmental concerns have been found to act as a positive mediator in the relationship between attitudes and purchase intentions, underscoring the pivotal role of ecological awareness in cultivating favorable attitudes towards organic food (Ahmed et al. 2021). Environmental concerns have also been shown to moderate the relationship between green preferences and purchase intentions, highlighting the importance of ecological values in shaping consumer behavior towards organic products (Ayaz and Jang 2024). Moreover, environmental concerns impact purchase behavior through attitudes, demonstrating their role in guiding decisions related to organic product purchases (Xing and Liao 2024; Bozkurt and Kocaadam‐Bozkurt 2024). Finally, studies found that environmental concerns exert a direct positive effect on purchase intentions, reinforcing their influence within the organic product context (Jin et al. 2024). Following this rationale, we propose the following hypothesis:

*Consumers' environmental concerns positively affect their attitudes towards organic foods*.

### 2.5 Consumer Attitudes

Consumer attitudes represent long‐term orientations in the minds of consumers and are one of the most important elements that shape consumer behavior (Ajzen 2018). While attitudes do not always translate directly into behaviors, such exceptions are relatively rare. In general, attitude‐behavior relationships tend to operate in the same direction also for healthy or sustainable food products (Vermeir and Verbeke 2008). Many studies have shown that a consumer's favorable attitude towards a food product significantly increases the likelihood of purchase (Le‐Anh and Nguyen‐To 2020; Aitken et al. 2020; Bazhan et al. 2024). Consistent with these findings, we expect that attitudes towards organic food will positively influence purchase intentions to purchase such products. Thus, we propose the following:

*Attitudes towards organic foods positively affect consumers' purchase intentions*.

### 2.6 Moderating Effects

One of the main objectives of this study is to address the organic attitude‐behavioral intention gap proposed by Massey et al. (2018) by examining moderators that could bridge this gap, as suggested by Liang and Lim (2020). We focus on two sets of moderating variables: trust and knowledge. The expected effects of these moderators are discussed in the following subsections.

#### 2.6.1 Trust in Organic Foods and Trust in Organic Labeling

Consumers need to have confidence in the claimed benefits of organic food and believe that the products they purchase and consume truly originate from a certified organic supply chain. Such confidence can significantly influence the formation of favorable attitudes and the likelihood of making a purchase among consumers (Cao et al. 2024). However, studies show that consumers are skeptical of claims about credence‐based food products such as organic and functional foods (Torres‐Ruiz et al. 2018; Arslan 2020). Trust is even more critical in emerging markets such as Turkey due to generally lower awareness and knowledge of organic products (Kushwah et al. 2019; Dinçer et al. 2023).

The effect of trust has been widely studied in organic consumer decision‐making studies. However, as noted, the fact that consumer trust is an abstract concept with multiple facets is somewhat neglected in the organic food literature (Zagata and Lostak 2012). Most of the studies subjected the concept to a relatively narrow perspective, focusing only on one side of the concept (Ashraf et al. 2018; Lee et al. 2020; Canova et al. 2020), although the literature suggests trust needs to be evaluated with a comprehensive perspective (Zagata and Lostak 2012; Nuttavuthisit and Thøgersen 2017). Trust in organic food involves trust in labeling/certification (Lee et al. 2020), trust in organic foods in general (Ashraf et al. 2018), as well as trust in retailers (Khare and Pandey 2017), and supply chains (Busch et al. 2024). The study discusses the dimensions of “general trust” and “trust in the certification process” in particular, which are considered to be more directly related to perceptions of the authenticity and credibility of organic foods. Both facets have been argued to influence consumers' organic food preferences (Nuttavuthisit and Thøgersen 2017), and this multi‐faceted trust suggested further investigation, especially in emerging markets (Truong et al. 2021). In this study, we therefore treat trust as a two‐dimensional construct: trust in organic foods and trust in organic labeling.

Previous studies also suggest the importance of examining trust in organic foods as a moderator, primarily to address the attitude‐behavioral intention gap in organic food studies (Lee and Yun 2015; Massey et al. 2018; Guo et al. 2021). In a recent study, Nguyen et al. (2023) suggested trust as a moderator between attitudes and purchase intentions, reasoning that trust strengthens positive reasons to buy organic food while decreasing the effects of negative factors that prevent purchase intentions. Accordingly, as a person's trust in organic products increases, the effect of positive attitudes on purchase intention can also be expected to increase. Taking into account these insights in the literature, we expect both kinds of trust to moderate the relationship between attitudes and purchase intentions. Thus, we propose:

*Consumer trust in organic foods moderates the relationship between attitude and purchase intentions, such that the relationship is stronger when trust in organic foods is higher*.

*Consumer trust in organic food labeling moderates the relationship between attitude and purchase intentions, such that the relationship is stronger when trust in labeling is higher*.

#### 2.6.2 Subjective and Objective Knowledge Towards Organic Foods

Consumers in emerging markets often struggle due to insufficient knowledge about organic products, certifications, and standards, which can negatively affect purchase intentions (Nautiyal and Lal 2025). Accurate knowledge about organic products is a key factor influencing their consumption, especially as it reduces consumer confusion (Dinçer et al. 2023). Despite the importance of “true” (objective) knowledge in consumer decision‐making, this aspect has been notably overlooked in efforts to address the attitude‐behavioral intention gap in organic food studies.

A lack of knowledge is one of the factors that complicates consumers' ability to distinguish between organic and conventional products (Gleim et al. 2013). According to Aertsens et al. (2009), enhancing customers' knowledge or providing them with more information about organic products can also be beneficial in reducing mistrust and doubts. Therefore, reducing uncertainty through increased information may, in turn, increase the likelihood of purchasing organic products (Thøgersen 2009). Indeed, some studies have found that an individual's lack of knowledge about organics can dampen purchase intentions, even if they hold positive attitudes (Thøgersen 2009). Furthermore, Nautiyal and Lal (2022) emphasized the role of objective knowledge as a facilitator of organic purchasing.

Subjective knowledge has also been considered an important concept in the relationship between attitude and behavioral intentions of organic foods. Brucks (1985) stated that “*How much an individual thinks they know about an alternative, what has been called ‘subjective knowledge’, may be related to how confidently they hold their attitude*”. Berger et al. (1994) posited that subjective knowledge is a significant determinant in product categories such as organic food. They proposed that attitudes would exhibit greater strength as subjective knowledge increases, consequently exerting a heightened influence on behavioral intentions. Thøgersen (2009) revealed subjective knowledge as a valid predictor in adapting eco‐labeled products and revealed that confusion and ignorance about organic products can negatively affect purchase intentions.

Both types of knowledge could potentially influence the translation of attitudes into purchase intentions. Consumers who believe they know much about organic products (high subjective knowledge) might be more confident in acting on their attitudes. However, if that confidence is misplaced (i.e., not backed by factual knowledge), it may not effectively drive behavior. Thus, examining both subjective and objective knowledge as moderators is important for a clearer understanding of the attitude‐intention link. We expect that higher knowledge will strengthen the impact of attitudes on purchase intentions. Therefore, we propose:

*Consumer subjective knowledge moderates the relationship between attitude and purchase intentions, such that this relationship is stronger at higher levels of subjective knowledge*.

*Consumer objective knowledge of organic food labeling moderates the relationship between attitude and purchase intentions, such that this relationship is stronger at higher levels of objective knowledge*.

**FIGURE 1:**
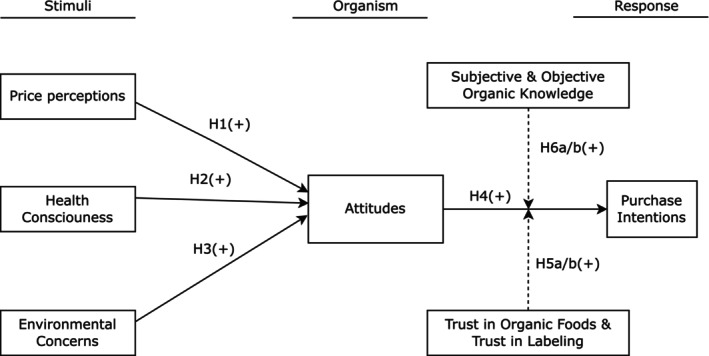
Research model.

Figure 1 illustrates the conceptual model consisting of the relationships mentioned above.

## 3 Method

### 3.1 Sampling and Data Collection Procedures

The data were collected using an online survey of Turkish consumers. The sampling frame consisted of individuals residing in Turkey, aged 18 and above, who had purchased organic food at least once in the past year. The convenience sampling method was used, with care taken to ensure maximum heterogeneity in order to make the sample as representative as possible of Turkish consumers. Participants were prevented from filling out the questionnaire more than once through IP restrictions. 552 questionnaires were obtained. The questionnaire used two control questions between the Likert scale statements (e.g., If you are reading this question, please tick the—strongly disagree—option on the Likert scale). We excluded 78 responses that failed these checks, leaving 474 valid questionnaires for analysis.

### 3.2 The Research Instrument

The questionnaire comprised three main sections. The first section provided a brief introduction, including details about the research purpose, the anonymity of the survey, and the estimated completion time. The second section featured Likert‐scale questions designed to assess the latent variables within the research model. The final section consisted of inquiries concerning the demographic attributes of the study sample.

### 3.3 Measurement

All the scales used in this study to measure the study constructs were drawn from previous research and have been pre‐validated (see Table 2). We used a 7‐point Likert scale to measure all scale items (1 = strongly disagree/7 = strongly agree) with the exception of the objective knowledge measure. We used four items from Tarkiainen and Sundqvist (2005) to measure health consciousness, five items to measure environmental concerns from Teng and Lu (2016), three items to measure price perceptions from Lee and Yun (2015), four items to measure attitudes from Ajzen (2001) and Arvola et al. (2008), and three items to measure purchase intention from Tarkiainen and Sundqvist (2005) and Pham et al. (2019). For the moderators, we used four items from Liang (2016) to measure trust in organic food labeling and six items from Anisimova et al. (2019) to measure trust in organic food. Subjective knowledge was measured with three items from Aertsens et al. (2011). Objective knowledge was assessed with an expanded version of the scale by Park et al. (1994), tailored to the context of organic food; it consisted of six true/false questions (scored as 1 for a correct answer, 0 for incorrect, for a total score ranging from 0 to 6).

## 4 Data Analysis and Results

### 4.1 Sample Characteristics

The demographic features of the study sample are presented in Table 1. The participants exhibit a heterogeneous structure in terms of income. While nearly 45% of the sample earns less than 30.000 TL, even the highest income group is adequately represented in the sample with 14.3%. Except for those over 61–70 years old, more than 65 people are in each age category, indicating a heterogeneous distribution. The ratio of female (54%) and male (46%) participants also represents a close distribution among the 474 survey participants. More than 61% of the participants have at least a bachelor's degree, while 29.3% have at most a high school degree. Lastly, while only 12.2% of the participants were living alone, 52.1% of them live in households of three or more people.

**TABLE 1:** Demographic features of respondents.

Demographic characteristics of the sample (*N* = 474)		Frequency	Percent
Income	17,000 TL and Below	111	23.4%
	17,001–30,000 TL	101	21.3%
	30,001–40,000 TL	53	11.2%
	40,001–50,000 TL	67	14.1%
	50,001–60,000 TL	74	15.6%
	60,001 TL and Above	68	14.3%
Age	15–20 years old	67	14.1%
	21–30 years old	97	20.5%
	31–40 years old	114	24.1%
	41–50 years old	111	23.4%
	51–60 years old	75	15.8%
	61–70 years old	10	2.1%
Gender	Male	218	46.0%
	Female	256	54.0%
Education	Primary and secondary school	12	2.5%
	High School	139	29.3%
	Undergraduate	33	7.0%
	Bachelor's degree	156	32.9%
	Postgraduate	134	28.3%
Living/w	Living Alone	58	12.2%
	With one person	70	14.8%
	With two people	99	20.9%
	With three people	117	24.7%
	With four people	76	16.0%
	More	54	11.4%

Abbreviation: TL, Turkish Lira.

### 4.2 Measurement Model

We conducted a confirmatory factor analysis (CFA) to evaluate the reliability and validity of the measures. As shown in Table 2, it is seen that the measurement model has a good fit, and all the values obtained were found to be within the recommended range in the literature: *χ* ^2^/df = 2.324, *p* < 0.01; RMSEA = 0.053; GFI = 0.923; CFI = 0.949; TLI = 0.939 (Hu and Bentler 1999; Hair et al. 2010). We evaluate the convergent validity following the suggestions of Hair et al. (2010). Accordingly, it is seen that the values we obtained both for CR (ranging from 0.83 to 0.94) and AVE (ranging from 0.63 to 0.81) were within the suggested ranges. Also, item loadings all exceeded 0.50, ranging from 0.71 to 0.91, which were statistically meaningful. Thus, it is seen that the results ensure convergent validity (Hair et al. 2010).

**TABLE 2:** Summary of the measurement model results.

Construct	Statements	FL
Attitude AVE = 0.81 CR = 0.94 *α* = 0.95	I think that purchasing organic food is a good idea	0.91
	I think that purchasing organic food is wise	0.91
	I think that purchasing organic food is important	0.89
	I think that purchasing organic food is beneficial	0.87
Health Consciousness AVE = 0.66 CR = 0.88 *α* = 0.89	I choose food carefully to ensure good health for myself	0.86
	I choose food carefully to ensure good health for my family	0.81
	I think of myself as a health‐conscious consumer	0.87
	I often think about health issues when buying food	0.82
Environmental Concerns AVE = 0.63 CR = 0.89 *α* = 0.88	Organic food is produced in a way that does not affect the balance of nature	0.71
	Organic food is packaged in an environmentally friendly manner	0.91
	Organic food products are produced without harming the environment	0.87
	Organic food is produced without causing pain to animals	0.79
	Organic food is produced while respecting animal rights	0.88
Perceived Price AVE = 0.72 CR = 0.84 *α* = 0.74	Organic food is expensive.	0.81
	The price of organic food is high	0.87
	Organic food product prices are reasonable (R)	0.77
Purchase Intention AVE = 0.66 CR = 0.85 *α* = 0.85	I would buy organic food instead of other foods if it is possible	0.82
	I will advise using organic food if asked	0.81
	I intend to buy organic food in the near future	0.78

Abbreviations: AVE, average variance extracted; CR, composite reliability; FL, factor loadings; *α*, Cronbach's alpha.

With regard to assessing discriminant validity, we adhered to the guidelines proposed by Fornell and Larcker (1981). As shown in Table 3, while the AVE values of constructs are larger than the MSV values, none of the AVE values were smaller than the square of latent variables' correlations, which ensures discriminant validity.

**TABLE 3:** Correlation matrix for the constructs of the study.

Correlation matrix	1	2	3	4	5	6	7	8	AVE	MSV
Price perceptions	1								0.72	0.03
2 Health consciousness	< 0.001	1							0.66	0.36
3 Environmental concerns	0.020	0.006	1						0.63	0.08
4 Attitudes	0.023	0.014	0.085	1					0.81	0.19
5 Purchase intentions	< 0.001	0.327	0.053	0.196	0.259	1			0.66	0.36

### 4.3 Main Effects in SEM

We used structural equation modeling to test the proposed effects represented in the research model. According to the results, the proposed research model had a good fit (*χ* ^*2*^ */df = 2.590, p* < 0.01; CFI = 0.954, TLI = 0.946, GFI = 0.914, RMSEA = 0.058). As represented in Table 4, all hypothesized direct effects were supported. H1 indicates that price perceptions towards organic foods significantly affect attitudes towards organic foods (*β* = 0.102, *p* < 0.05). Health consciousness had a significant effect on attitudes (*β* = 0.074, *p* < 0.05), supporting the hypothesis H2. Environmental concerns likewise showed a significant effect on attitudes (*β* = 0.415, *p* < 0.01), thus supporting H3. Lastly, attitudes exhibited a strong positive relationship with purchase intentions (*β* = 0.689, *p* < 0.01), which supports H4.

**TABLE 4:** Structural model hypothesis test results.

Hypotheses	*β*	*t*‐value	Result
*Direct effects*			
H1	0.10**	2.94	Supported
H2	0.07**	2.73	Supported
H3	0.42***	5.48	Supported
H4	0.68***	8.10	Supported
*Moderating effects*			
H5a	0.13**	2.26	Supported
H5b	0.12**	2.19	Supported
H6a	0.07	1.23	Not supported
H6b	0.15***	2.76	Supported

** *p* < 0.05.

*** *p* < 0.01.

### 4.4 Moderation Analysis Results

We examined the moderating effects of trust and knowledge using Hayes' PROCESS macro with bootstrap confidence intervals (Hayes et al. 2017). Specifically, we applied Model 1, which is designed to test one moderator at a time. Since our study includes multiple moderators, this approach further helps to reduce potential multicollinearity, as each moderator is analyzed in a separate model rather than simultaneously. The analysis was conducted using 5000 bootstrap samples with 95% confidence intervals. To further minimize potential multicollinearity, all continuous variables were mean‐centered prior to the creation of interaction terms (Aiken and West 1991).

The results indicated that trust in organic foods significantly moderates the relationship between attitude and purchase intention, which supports H5a. The interaction term for attitude × trust in organic food was significant (*F*_*[1,470]*_ = 5.14, *ΔR* ^2^ = 0.009, *β* = 0.13, *p* < 0.05). This indicates that when consumers have higher trust in organic foods, the influence of their attitude on their purchase intention becomes stronger. Similarly, trust in organic labeling also significantly moderates the relationship between attitude and purchase intention (*F*_*[1,470]*_ = 4.80, *ΔR* ^2^ = 0.009, *β* = 0.12, *p* < 0.05). Accordingly, H5b was supported.

For the knowledge moderators, objective knowledge significantly moderated the attitude–purchase intention link (H6b: *F*_*[1,470]*_ = 7.52, *ΔR* ^2^ = 0.012, *β* = 0.15, *p* < 0.01). Consumers with greater factual knowledge about organics exhibited a stronger translation of attitude into purchase intention. However, subjective knowledge did not show a significant moderating effect (H6a was not supported, *p* > 0.05). This suggests that merely feeling knowledgeable (without actual, accurate knowledge) did not amplify the attitude–intention relationship in our sample.

## 5 Discussion

This study aimed to explore Turkish consumers' intentions to purchase organic food using the S‐O‐R model as a theoretical lens. SEM results with 474 respondents' data supported most of the hypothesized relationships.

For H1, we found a positive effect of price perceptions on attitudes, which supported the first hypothesis. This finding contradicts earlier studies reporting a negative relationship between price and attitudes (Fotopoulos and Krystallis 2002; Pellegrini and Farinello 2009), yet aligns with a more recent meta‐study suggesting that price may be interpreted as a signal of quality or status (Massey et al. 2018). This may also reflect consumer hedonism, where premium pricing enhances perceived value and emotional satisfaction (Lee and Yun 2015). This phenomenon has been noted among health‐conscious hedonists (Tivadar and Luthar 2005; Morais et al. 2024), who derive satisfaction from purchasing premium, healthy products.

Our findings also supported H2, which proposed consumer health consciousness as a stimulus to attitudes. This is consistent with earlier findings arguing that health‐conscious consumers create more positive attitudes towards organic food (Mai and Hoffmann 2012; Irianto 2015; Bazhan et al. 2024; Hu et al. 2024). It reinforces the idea that consumers who prioritize health tend to appreciate the benefits of organic products, thereby developing favorable attitudes.

H3 was also confirmed, which proposed environmental concerns as a stimulus to attitudes. This mirrors results from previous research emphasizing environmental motivation as a strong driver of organic attitudes (Padel and Foster 2005; Liang and Lim 2020; Hu et al. 2024; Xing and Liao 2024). Our findings support the idea that environmental concern leads to positive attitudes in this context, consistent with studies such as Yadav and Pathak (2016) and Eberle et al. (2022). While some studies have found weak or no effects (e.g., Pham et al. 2019; Le‐Anh and Nguyen‐To 2020), our results affirm its relevance in the Turkish context.

H4 proposed a positive relationship between organic food attitudes and purchase intentions, which, too, was supported by our analysis while at the same time confirming previous literature in the organic food field (Lee and Yun 2015; Liang 2016; Le‐Anh and Nguyen‐To 2020; Aitken et al. 2020). Although some previous studies have found a weak or even negative attitude‐behavior relationship (Pellegrini and Farinello 2009; Lee and Yun 2015) or no relationship (Hempel and Hamm 2016), our findings are more consistent with the prevailing evidence that attitudes are a significant predictor of behavioral intentions. This consistency reinforces the validity of the attitude‐intention link in the context of organic food, particularly when attitudes are shaped by strong health and environmental motivations.

To deepen our understanding of the attitude–intention relationship, we tested whether trust and knowledge moderate this link, each conceptualized in two dimensions. Three of the four moderation hypotheses were supported, highlighting the critical role of knowledge and trust in bridging the attitude–behavioral intention gap. Although recent literature suggests a vital gap between organic attitudes and behavioral intentions (Massey et al. 2018), it is noteworthy that there is a very limited number of studies suggesting subjective or objective organic knowledge to address this gap, and there is no study investigating these moderator effects of different dimensions of trust and knowledge at the same time. One of the primary purposes of this study was to address this important gap.

Accordingly, the analysis revealed that objective knowledge (factual knowledge about organic food) significantly moderated the relationship between attitude and purchase intentions of organic foods (H6b). Thøgersen (2009) indirectly supported this finding by suggesting that a lack of organic knowledge would negatively affect purchase intentions even if there are positive attitudes. Teng and Lu (2016) also suggested uncertainty of organic products as a significant moderator, which is closely related to objective knowledge. In a recent study, Nautiyal and Lal (2022) propose objective knowledge as a moderator between attitudes and purchase intentions of organic food. The findings obtained within the scope of our study support the findings of these few studies in the literature. The results of our study contributed to the existing body of knowledge by supporting this finding, which is quite rare in the literature. This empirically supports the idea that actual knowledge about organic products acts as a driving force in increasing consumers' propensity to purchase organic products.

Contrary to our expectations, no significant moderating effect was found for subjective knowledge (H6a). This divergence is intriguing and suggests that what consumers actually know is more important than what they think they know when it comes to acting on their attitudes. As subjective knowledge is mainly treated as an antecedent to attitudes or purchase intentions in organic literature (i.e., Lee et al. 2020; Pacho 2020), there are few studies directly comparable to our findings, reinforcing the distinct role of objective knowledge in bridging the attitude–intention gap. Accordingly, in their studies, both Brucks (1985) and Berger et al. (1994) stated that the strength of attitudes would increase as subjective knowledge increased, hence having a greater impact on behavioral intentions. Hidalgo‐Baz et al. (2017) also proposed subjective knowledge as a significant moderator between organic attitudes and purchase intentions. They claimed that subjective knowledge shrinks the attitude–behavioral intention gap. Our findings do not align with those studies reported in the existing literature, which lacks a well‐established consensus. This divergence can be regarded as a significant contribution to the body of research on the relationship between organic attitudes and behavioral intentions.

Both trust dimensions, trust in organic foods and trust in organic food labeling, were revealed to moderate the relationship between attitude and purchase intention positively and significantly, which supports H5a/H5b. Thus, as consumers' trust in organic products and certification processes increases, the effect of consumer attitudes on purchase intention will become stronger. This finding is compatible with the rare studies in the literature. Guo et al. (2021) also demonstrated the moderator effect of trust between nutritional info and willingness to pay. Furthermore, Truong et al. (2021) revealed distrust in the organic food system as an important barrier to consuming organic food products. Considering the recommendation of Lee and Yun (2015) to consider trust as a moderator of organic product purchasing behavior, it can be said that our study is an important contribution to the literature because the literature has not tested whether there is a multidimensional moderator effect specifically among our variables. Our study is the first to do so.

This is particularly relevant for emerging markets like Turkey, where skepticism about certification and regulatory systems may limit organic adoption. Strengthening consumer trust could therefore be essential for narrowing the attitude–intention gap.

Overall, our findings reinforce the applicability of the S‐O‐R framework to the organic food context and highlight that integrating multidimensional trust and knowledge helps explain the attitude–intention gap identified in prior research.

## 6 Conclusions

Unlike many developed markets, emerging economies have received limited scholarly attention concerning the antecedents of organic food purchase intentions within robust theoretical frameworks. Addressing this gap, the present study examined Turkish consumers through an empirically tested model grounded in the Stimulus–Organism–Response (S‐O‐R) framework, an approach that adds conceptual depth to the understanding of sustainable food choices in transitional markets.

Moving beyond a narrow focus on attitude formation, this study engaged with the widely acknowledged attitude–behavior discrepancy by proposing a multidimensional model that not only identifies the key antecedents of consumer attitudes but also explores the contextual conditions under which these attitudes more effectively translate into behavioral intentions. In this way, the research contributes to the literature by enhancing existing models both in terms of structural breadth and moderating complexity.

The model incorporated three stimulus factors—price perceptions, health consciousness, and environmental concerns (S); attitudes as the organism‐level evaluation (O); and purchase intention as the response behavior (R). Furthermore, four moderators, trust (in both product and labeling) and knowledge (both subjective and objective), were introduced to account for the variability in the strength of the attitude–intention relationship.

Empirical analyses confirmed all hypothesized direct effects and revealed significant moderating effects for trust and objective knowledge. Specifically, the likelihood of attitudes converting into intentions increases when higher levels of trust and accurate knowledge are present. These findings provide timely insights into the boundary conditions that shape intention formation, particularly in emerging markets. Rather than treating trust and knowledge as peripheral influences, the study positions them as pivotal mechanisms for narrowing the persistent attitude–behavior gap in organic food research. In contexts such as Turkey, where institutional trust and consumer awareness are relatively limited, these insights are especially relevant, underscoring the importance of credible certification systems and knowledge‐enhancing interventions to support organic consumption.

By differentiating between types of trust and knowledge, the model achieves high explanatory power and contributes a stronger understanding of consumer intention formation. In doing so, the study expands the empirical application of the S‐O‐R framework and offers a refined analytical tool to the sustainable consumption literature. Although the data were collected in Turkey, the framework and findings offer broader implications for similar emerging or transitional markets.

Overall, this research contributes a theoretically grounded and empirically validated model that not only identifies key drivers of organic food purchase intentions but also illuminates how trust and knowledge interact with consumer attitudes to shape behavioral outcomes. It thus offers a timely and multidimensional contribution to the academic and practical discourse on organic food adoption. Building on these, the following section outlines the theoretical and managerial implications that arise from this study.

## 7 Implications

### 7.1 Theoretical Implications

This study contributes to theory in several important ways. First, by adapting the S‐O‐R framework in the context of organic food consumption, this research provides a comprehensive explanation of organic food purchase intentions, validating the model in the context of an emerging market. Unlike previous studies, which have tended to focus on either attitudes or intentions in isolation, our model integrates both levels alongside external stimuli and moderators. This offers a more complete view of the psychological processes behind decisions to purchase organic food.

Second, it provides novel insights into the moderating roles of bi‐dimensional trust and dual aspects of knowledge in bridging the gap between attitudes and purchase intentions. By differentiating between trust in organic products and in labeling, and between subjective and objective knowledge, the study advances the understanding of consumer behavior in emerging markets where trust in regulatory systems and organic knowledge is often limited. This dual conceptualization also refines the Organism–Response link in the S‐O‐R model, offering a more nuanced theoretical lens to explore the widely acknowledged attitude–intention gap.

Finally, the study addresses both geographical and theoretical gaps by focusing on Turkey, which is an underrepresented emerging market in sustainability research. The findings show that structural models developed in more established markets need to be adapted to local conditions to remain valid. This offers a framework that can be replicated in future studies of similar socio‐economic environments and adds contextual depth to the sustainable consumption literature.

### 7.2 Managerial Implications

The results revealed in this study have particular reference value for both policymakers and practitioners, seeking to grow the organic food market in Turkey and similar emerging markets. Turkey's annual per capita expenditure on organic food (around €1) is very low, indicating that the domestic organic market has significant room for growth (Turkish Ministry of Food Agriculture and Livestock 2013–2016 Organic Agriculture National Action Plan). Our findings can guide stakeholders in designing strategies to stimulate this growth.

First, since consumer attitudes emerged as a critical predictor in our study, it is crucial for the authorities to plan and implement better strategies to create more positive attitudes towards organic foods. Public awareness campaigns and educational programs highlighting the health and environmental benefits of organic food products can be effective in this regard. By increasing consumers' awareness of the personal and societal advantages of organic consumption (e.g., healthier lifestyles, environmental protection), such initiatives can foster more favorable attitudes and greater willingness to buy organic.

Second, trust emerged as a key factor moderating the attitude‐intention relationship. This underscores the need for efforts to increase consumers' trust in organic foods and labels. Our sample indicated only moderate trust levels on average (mean “trust in organic food” = 4.11 and “trust in labeling” = 4.28 out of 7). Engaging in practices that will increase consumers' trust in organic foods, such as ensuring credible certification and transparency, has the potential to play an essential role in boosting domestic consumption. Consumer trust also seems like one of the most crucial issues for practitioners as well. This situation becomes even more critical given that trust acts as a moderator role in the relationship between attitudes towards organic products and purchase intentions. Turkish retailers must find ways to overcome this trust issue. In this sense, it may be beneficial to implement stricter regulations against retailers who improperly use organic labels. A well‐organized supply chain in the Turkish organic food industry, improved transparency within organic supply chains, and the adoption of digitally secure and trackable labeling can be instrumental in addressing the trust deficit and ultimately strengthening consumer trust and positive attitudes towards organic food in Turkey.

Third, objective knowledge significantly enhances the translation of attitudes into purchase intentions, whereas subjective knowledge does not. This suggests that consumers need access to verifiable, accurate information rather than simply feeling informed. Policymakers and retailers should invest in public awareness programs, in‐store explanations, labeling clarity, and community‐based outreach to improve factual knowledge.

Lastly, although our results show that the high price of organic food may improve perceived quality and attitudes for some consumers, affordability remains a barrier for many in emerging markets. Policymakers can take initiatives to make organic products more accessible without undermining their premium image. For example, providing subsidies or financial incentives to local organic farmers can reduce production costs, thereby lowering retail prices and widening consumer access.

In summary, in order to stimulate the growth of the organic market in Turkey, stakeholders should focus on fostering positive attitudes (by raising awareness of health and environmental benefits), building trust (by ensuring transparency and credible certification), improving objective knowledge (by providing clear information and education), and carefully addressing affordability (by implementing supportive policies) without compromising perceived quality. Putting these strategies into practice can help convert more customers who are ‘interested’ in organic products into loyal buyers.

### 7.3 Limitations and Future Research

Firstly, we asked participants' thoughts regarding organic foods in general instead of using a product category. This broad approach might overlook product‐specific nuances. Using a specific product category (e.g., organic fruits or dairy) in future studies could provide more specific insights. Secondly, the data were self‐reported, which may have affected participants' responses due to reasons such as social desirability bias. Future research could incorporate measures to detect or mitigate social desirability bias (e.g., including a social desirability scale or using indirect questioning techniques).

Thirdly, the study collected data from Turkish consumers using non‐probability convenience sampling. This restricts the generalizability of the findings. The sample may not be representative of the general population, and focusing on only one country limits the applicability of the results to different cultural contexts. To increase external validity, future studies are recommended to use probability‐based sampling methods or examine consumers in different regions and countries.

Finally, this study adopted a cross‐sectional design, measuring consumer perceptions at only one point in time. This makes it difficult to observe changes over time or establish causal relationships. Future longitudinal research tracking how attitudes and purchase intentions evolve over time could provide deeper insights and more robust practical implications.

## Author Contributions

**Yusuf Arslan:** conceptualization (lead), data curation (lead), formal analysis (lead), funding acquisition (lead), investigation (lead), methodology (lead), project administration (lead), resources (lead), software (lead), supervision (lead), validation (lead), visualization (lead), writing – original draft (lead), writing – review and editing (lead).

## Conflicts of Interest

The author declares no conflicts of interest.

## Data Availability

The data that support the findings of this study are available from the corresponding author upon reasonable request.
